# Supplementary figures and images for: A multifaceted role of progranulin in regulating amyloid-beta dynamics and responses
Source: Life Sci Alliance. 2021 Jun 8;4(7):e202000874. doi: 10.26508/lsa.202000874 (PMC8200295; doi:10.26508/lsa.202000874)

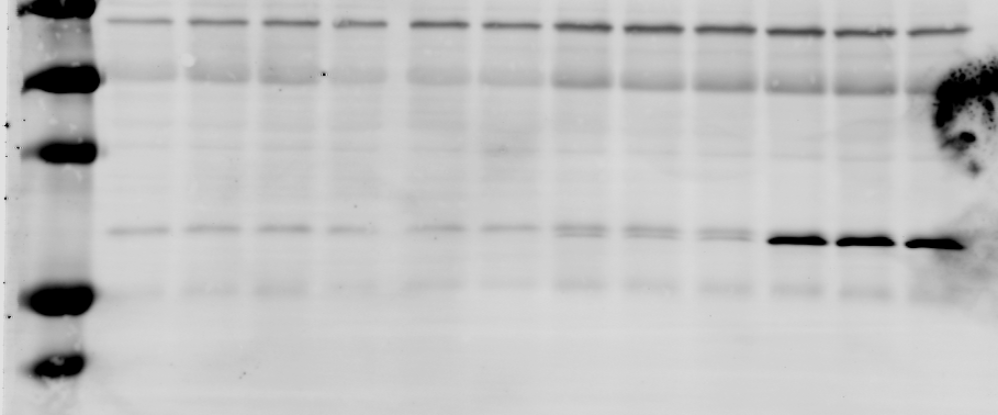

Supplement: Supplementary file 2 [file LSA-2020-00874_SdataF4.tif]
